# A Novel Approach for Stereotactic Radiosurgery of Multiple Brain Metastasis in a Patient With Poor Performance Status: A Case Report

**DOI:** 10.1155/crom/3420915

**Published:** 2025-12-22

**Authors:** Arad Iranmehr, Shakiba Sheikholeslami, Nooshin Banaee, Mohammad Shirani, Mostafa Farzin, Fatemeh Jafari

**Affiliations:** ^1^ Neurosurgery Department, Sina Hospital, Tehran University of Medical Sciences, Tehran, Iran, tums.ac.ir; ^2^ Gamma Knife Center, Yas Hospital, Tehran University of Medical Sciences, Tehran, Iran, tums.ac.ir; ^3^ Radiation Oncology Department, Cancer Institute, Imam-Khomeini Hospital Complex, Tehran University of Medical Sciences, Tehran, Iran, tums.ac.ir; ^4^ Department of Nuclear Engineering, CT.C, Islamic Azad University, Tehran, Iran, azad.ac.ir

**Keywords:** brain metastases, radiosurgery, stereotactic radiosurgery

## Abstract

**Background:**

Brain metastases are the most common malignant intracranial tumors in adults. The management of patients with poor performance status and multiple brain metastases remains controversial, with guidelines generally recommending palliative care in such cases. Stereotactic radiosurgery has been debated for this group of patients, particularly those with multiple lesions.

**Case Presentation:**

We present a case of an Iranian patient with metastatic non‐small cell lung cancer (NSCLC) who had previously undergone whole‐brain irradiation (WBRT). The patient was referred due to the progression of multiple brain lesions. In response, we administered two separate courses of fractionated stereotactic radiation therapy (FSRT) to treat these brain masses. The patient′s neurological symptoms showed significant improvement following the treatment.

**Conclusions:**

This case suggests that stereotactic radiation therapy can be considered for patients with poor performance status, regardless of the number of brain metastases. It may stabilize or improve neurological deficits and enhance the patient′s quality of life, even if its effect on overall survival is uncertain. Additionally, treating the lesions in two separate courses may reduce treatment time and facilitate better repair of normal brain tissue.

## 1. Introduction

Brain metastases are the most common type of brain tumors in adults. An estimated 98,000–170,000 cases occur annually in the United States. The incidence of brain metastases is increasing due to several factors [1] Patients with metastatic disease have a longer survival with new systemic therapies (including immunotherapy) that have recently seen more widespread use. Furthermore, the growing use of sensitive magnetic resonance imaging (MRI) techniques has contributed to better detection of small asymptomatic lesions [2]. Improving radiation and surgical techniques and developing stereotactic radiation, the treatment of brain metastases is becoming individualized based on patient performance, number of brain lesions, extracranial status, histology, and estimated surveillance. Treatment options include surgery, stereotactic radiosurgery (SRS), whole‐brain irradiation (WBRT), and best palliative care.

Recent ASCO–SNO–ASTRO and NCCN guidelines recommend SRS for one–four brain lesions and suggest WBRT for patients with more than four lesions if local therapy is indicated. Guidelines almost always recommend the best palliative care for poor performance [3].

Obviously, patients who become candidates for the best palliative care experience progressive neurological deficits and worsening quality of life until the time of death. A few studies have investigated stereotactic radiation therapy (SRT) in patients with poor performance [4–6], and no study has investigated SRT′s usefulness in patients with poor performance and multiple brain metastases. Here, we discuss a single patient with multiple brain metastases whose extracranial tumor was controlled and whose performance status worsened due to intracranial progression.

We treated our patient with fractionated stereotactic radiation (FSRT) and, to overcome the treatment limitations for this patient, followed some logistical solutions.

## 2. Case Presentation

A 48‐year‐old nonsmoker Iranian woman complained of a cervical mass on the left side of her neck. She was diagnosed with lung adenocarcinoma in July 2022. The primary lesion was approximately 3 cm in the central portion of the left lung upper lobe, with minimal pleural effusion. Pathologic lymphadenopathies were detected in the left jugular chain. Screening MRI was performed, which demonstrated multiple brain metastases with five lesions involving both hemispheres. There were no other metastatic lesions in other parts of the body. As a molecular pattern, the tumor exhibited EX19Del on the EGFR gene and did not express PDL1. KRAS and BRAF mutation and ROS1 rearrangement were not detected.

The patient initially received six courses of chemotherapy with carboplatin and paclitaxel. After the third course of chemotherapy, due to the unavailability of stereotactic radiotherapy techniques in the patient′s home city, she was treated with WBRT (30 Gy in 10 fractions). At the end of the six courses of chemotherapy, the cervical lymph nodes disappeared, and the primary lesion remained stable in size. Subsequently, erlotinib was initiated as a maintenance therapy. Three months after the erlotinib initiation, she complained of shortness of breath, and the disease progressed to severe pleural effusion. Pleurectomy was performed, and the treatment was changed to cisplatin and vinorelbine.

There was no evidence of disease progression for approximately 6 months, and her performance status was 90 on the Karnofsky Performance Scale (KPS) and 0 on the Eastern Cooperative Oncology Group (ECOG) scale. Six months later, the patient gradually experienced imbalance, abnormal gait, memory loss, and sensory/motor dysfunction in four limbs, especially in the upper left one. A brain MRI was performed, and 42 metastatic intracerebral lesions were detected. The size, number, and peripheral edema around the lesions increased significantly compared with previous MRI findings. Her performance status was 50 on KPS and 3 on the ECOG scale. The whole‐body FDG‐PET scan was done; no other metastatic lesion was found, and the primary lung lesion was stable.

As extracranial lesions were stable during the last 6 months and only intracranial progression influenced her performance, we decided to treat the brain metastases locally rather than with palliative care. Since the patient had received WBRT about 10 months ago and not many studies support whole‐brain reirradiation, she would probably experience neurocognitive deterioration by whole‐brain reirradiation, which would affect her quality of life. Due to the number of metastases located near the hippocampus, we were unable to perform hippocampus‐avoidance WBRT.

**Figure 1:**
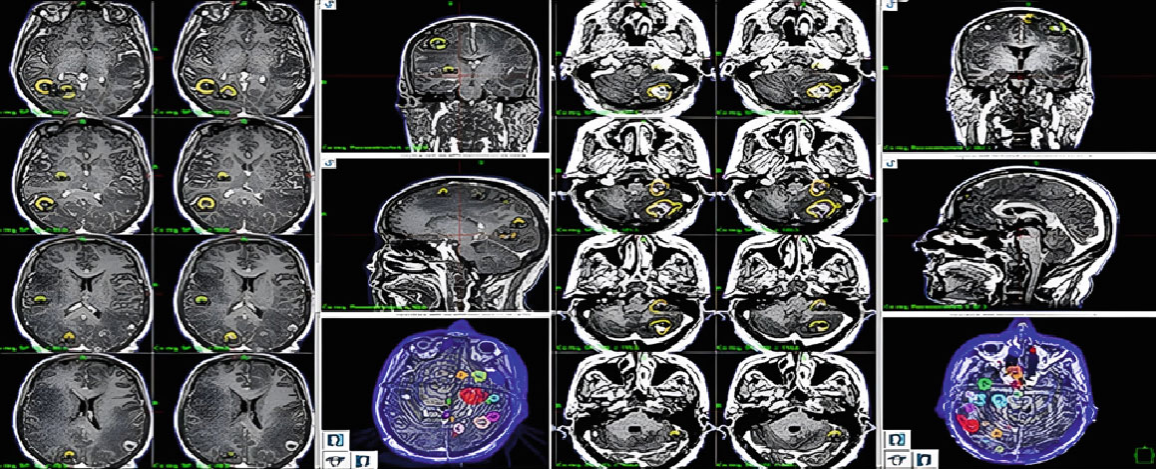
Delineated targets in each hemisphere.

We treated brain lesions with stereotactic radiation and chose FSRT instead. A spiral brain CT scan without contrast (slices of 1 mm thickness) was performed, and a brain gadolinium‐enhanced MRI (1 mm slice thickness) was fused to the simulation CT scan for brain metastasis detection. A radiation oncologist and neurosurgeon delineated all metastatic lesions separately on T1 enhanced MRI as GTV, and no extra margin for PTV was added. Peripheral edema was not included in the target volume. Eighteen brain metastases in the right cerebral hemisphere and 24 in the left cerebral hemisphere were identified (shown in Figure 1). Our primary goal was to treat all lesions in one course of FSRT (5 Gy in five sessions), but after treatment planned by a radiation physicist, we faced some limitations. First, treating multiple brain metastases in a single session would have required more than 3 hours of continuous immobilization. Such prolonged treatment could be particularly challenging for patients with poor performance status, given the need to remain fixed in the stereotactic head frame throughout the entire procedure. Second, we were concerned about the potential for acute radiation‐induced edema and radionecrosis. A prior history of WBRT and a high cumulative intracranial tumor volume were known risk factors for adverse radiation effects (ARE) [7], both of which were present in our patient. In addition, we were not certain how much benefit the treatment would ultimately provide, and we were concerned about the possibility of overtreatment.

Our team of radiation oncologists, a neurosurgeon, and a medical physicist discussed the case and decided to initiate treatment of the patient′s right hemispheric lesions with FSRT (25 Gy in 5 fractions). Although the number of lesions in the left hemisphere was higher, most of the edema occurred on the right side, and the patient′s symptoms were consistent with the right‐sided lesions. If the treatment had been helpful and the patient′s status was kept stable or improved, the left hemisphere brain lesions would be treated with another course of FSRT after 2 weeks. By treating the lesions that caused more symptoms first, we probably had a better chance of achieving clinical improvement. We supposed that creating such a sequence in treatment could be beneficial in several ways. First, by evaluating early clinical responses after the initial course, we could decide about other lesion treatments and avoid possible overtreatment; therefore, if FSRT was not helpful, the treatment could be interrupted. Second, the normal brain tissue receives less radiation in the first treatment course and could be repaired within 2 weeks before the subsequent treatment begins, which could help control brain edema and its clinical symptoms. Third, by splitting the treatment and reducing the time of each session, we would be able to make the treatment more comfortable and feasible.

**Figure 2:**
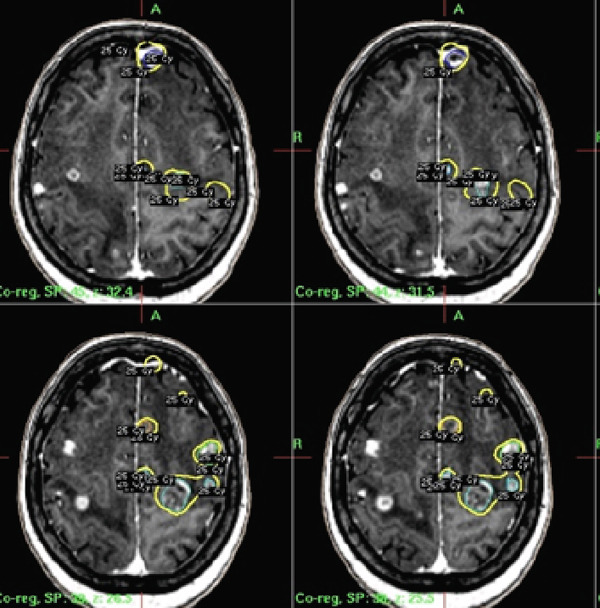
Dose distribution shown on four axial slices around the delineated targets. The yellow lines represent the 25 Gy prescription, corresponding to the 50% isodose line.

After explaining the advantages and disadvantages of the available treatments to the patient and family members, the patient underwent gamma knife stereotactic radiation on December 30, 2023. The first course of FSRT was initiated with 25 Gy in 5 fractions to the right hemisphere, which comprised 18 lesions, followed by treatment of the left hemisphere lesions, which included 24 lesions. The cumulative volume irradiated in the first course was 16.148 cm^3^, with the largest lesion measuring 3.505 cm^3^, and in the subsequent radiation course the total target volume was 18.938 cm^3^, with the largest lesion measuring 3.060 cm^3^. The marginal isodose was 50%, and a marginal dose of 25 Gy was prescribed to the 50% isodose line encompassing the tumor (shown in Figure 2). Due to the diffused spatial distribution of the lesions, it was necessary to use several matrixes. As one of the critical considerations of this plan, it was tried to spare normal tissues of the brain as much as possible, which caused using several small collimators (4 and 8 mm). A daily dose of 4 mg dexamethasone was prescribed a day before FSRT initiation, continued for a week during the first treatment course, and then tapered gradually. One week after the first course of treatment, we re‐evaluated the patient. There were no signs or symptoms of early ARE such as progressive headache, nausea, or neurological deficits. There was a slight improvement in left limb force and imbalance. A second course of FSRT for left‐hemisphere lesions was initiated 2 weeks later, similar to the right. During this time, systemic therapy with cisplatin and vinorelbine was continued.

We followed up our patient for 1 and 3 months after the local brain treatment. The patient′s motor force, balance, and performance status improved significantly. She entered the medical office by herself with a walker, was able to take care of herself, and carried out limited activities at home. We evaluated our patient with KPS of 70 at that time. A new MRI was performed 3 months after radiation completion, and all lesions were stable or had disappeared; however, two new metastatic lesions appeared in the right frontal lobe, and we referred her to her medical oncologist to decide about her systemic treatment.

## 3. Discussion

Currently, SRS is considered a key treatment for patients with fewer than five metastatic brain lesions, and WBRT is selected for patients with more than 10 lesions. However, it is sometimes difficult to use WBRT. First, WBRT can cause neurocognitive impairment, especially in patients who may live longer than a few months. Second, many patients refuse or dislike WBRT owing to neurotoxicity. Third, because of the improved patient survival, more patients who are irradiated for brain metastases develop intracerebral recurrences, requiring subsequent courses of radiotherapy. Many patients, as in our case, have synchronous brain metastases and have received WBRT during their disease presentation. Most centers are hesitant about reirradiation with WBRT because not many studies support it. In the five studies investigating whole‐brain reirradiation, encephalopathy and neurological decline were reported in two of these studies [8].

Multifraction SRS has been used as an alternative to single‐fraction SRS to reduce the incidence of radiation‐induced toxicity while maintaining high local control due to the high alpha‐to‐beta ratio in brain metastases in comparison to normal brain tissue, especially in non‐small cell carcinoma metastases [9, 10].

Despite the increased use of stereotactic radiation for multiple brain metastases, these patients have almost always been excluded from studies. The risk of microscopic metastases that are not visible on imaging increases as the number of metastases increases. Depriving patients of WBRT may increase the risk of new intracranial lesions appearing after SRS. The total dose of spillage and the integral dose to the entire normal brain tissue increase because of the sizeable cumulative target volume in multiple brain metastases [11].

Becker et al. estimated the maximum number of BM that can be treated with SRS or FSRT, while limiting the radiation dose delivered to normal brain tissue to that associated with WBRT. Their results suggested that treating 12–13 tumors per day over 10 days would deliver the dose of radiation to healthy brain tissue typically associated with a standard course of WBRT [12].

Traditionally, treatment of brain metastasis has been reserved for patients with good performance status, and patients with poor performance status often receive the best palliative care. Aggressive therapy for brain metastases in patients with poor performance status is not warranted. The only prospective data that compromised WBRT to best palliative care were from the council trial (Quartz trial), in which, in this study, there was no difference in overall survival and quality‐adjusted life years between these two groups [13].

No study is available that compares SRS or FSRT to the best supportive care in patients with poor performance. Untreated intracranial metastatic disease is generally associated with neurological deterioration, with consequent effects on quality of life and even death. Harat et al. in a retrospective analysis of poor‐performance patients with brain metastasis showed that SRS in patients with neurological symptoms could provide neurological improvement. More than 50% of the symptomatic patients in this analysis were stable or improved 3 months after SRS [6]. Kubicek et al. postulated that SRS for brain metastases in patients with poor performance status may improve their quality of life with an acceptable risk of new metastases, which increases overtime and may show the benefit of SRS even with low survival time [4]. In a retrospective cohort, Holub et al. showed that patients with brain metastasis and poor performance, especially if treated simultaneously with systemic therapy, may have a reasonable survival benefit after SRT [14].

Nevertheless, some serious issues should not be neglected in stereotactic radiation or FSRT of multiple brain metastases. First, the time of treatment may be long owing to the large number of brain metastases, which can affect tumor control and patient comfort. Second, the total spillage and energy leakage dose should be considered for large target volumes [11]. By dividing our treatment into two separate courses of FSRT for each cerebral hemisphere lesion, we reduced the treatment time, provided better motion control, and provided time for normal brain tissue repair.

As the patient′s performance and neurocognitive condition are not expected to improve with best palliative care, using stereotactic radiation could be considered a treatment for poor performance patients regardless of the number of brain masses, aimed at stabilizing or improving neurological deficits and quality of life, regardless of its effect on surveillance. For better motion control, decreased treatment time, and reduced radiation impact on normal brain tissue, dividing the treatment into courses may be helpful.

## 4. Conclusion

In the advent of SRS and FSRT, brain metastases treatment has been revolutionized, especially for patients with a limited number of metastatic lesions or not being candidates for WBRT due to cognitive decline risks or prior history of WBRT. Although WBRT continues to play an important role in multiple brain metastases treatments, its drawbacks, including cognitive neurotoxicity as well as reirradiation challenges, have led to increased interest in SRS and FSRT as potential alternatives. Nevertheless, treating multiple brain metastases with SRS or FSRT comes with its own set of challenges, which radionecrosis is at the most level of importance.

For our patient with poor performance status, FSRT in two divided treatment courses has shown promise in improving neurological symptoms and quality of life, even in the absence of significant survival benefits. This underscores the importance of considering stereotactic radiation not only for its potential to extend survival in a curative setting but also for its ability to enhance neurological function and overall quality of life. With current advances in systemic disease control, the traditional approach of assigning patients with multiple brain metastases solely to palliative care may warrant re‐evaluation. We suggest that stereotactic radiotherapy could be a reasonable option for carefully selected patients whose neurological symptoms are primarily due to intracranial disease and whose extracranial disease remains controlled, regardless of the number of lesions. Techniques such as splitting the treatment course could help overcome planning and feasibility limitations. Future studies with larger cohorts and prospective designs are needed to clarify optimal patient selection and to quantify the benefits of this approach.

## Ethics Statement

This study concurred with the Declaration of Helsinki. As stereotactic radiosurgery is a standard treatment modality in brain metastases, formal institutional review board approval was waived.

## Consent

Written informed consent was obtained from the patient for publication of this case report and any accompanying images. A copy of the written consent is available for review by the editor in chief of this journal.

## Conflicts of Interest

The authors declare no conflicts of interest.

## Author Contributions

Arad Iranmehr: data curation, methodology, supervision, and writing—original draft. Shakiba Sheikholeslami: data curation, methodology, and writing—original draft. Nooshin Banaee: methodology and writing—review and editing. Mohammad Shirani: resources and writing—review and editing. Mostafa Farzin: resources and writing—review and editing. Fateme Jafari: data curation, investigation, methodology, project administration, supervision, writing—original draft, and writing—review and editing.

## Funding

No funding was received for this manuscript.

## Data Availability

The authors confirm that the data supporting the findings of this case report are available within the article.
